# Supplementary material for: Genome-Wide Identification and Analysis of Chitinase GH18 Gene Family in Trichoderma longibrachiatum T6 Strain: Insights into Biocontrol of Heterodera avenae
Source: J Fungi (Basel). 2025 Oct 1;11(10):714. doi: 10.3390/jof11100714 (PMC12565608; doi:10.3390/jof11100714)
Supplement: Supplementary file 1 [file jof-11-00714-s001.zip › jof-3841329-GH18 Table S1.pdf]

Table S1 Bioinformatics analysis websites in this study.

Software names	Access time	Online website
NCBI database	24 April 2025	http://www.ncbi.nlm.nih.gov/
Pfam database	24 April 2025	http://pfam.xfam.org/
SMART database	24 April 2025	http://smart.embl-heidelberg.de/
ExPASy	24 April 2025	https://web.expasy.org/compute pi/
WoLF PSORT	24 April 2025	https://wolfpsort.hgc.jp/
SignalP-5.0	25 April 2025	https://services.healthtech.dtu.dk/service.php?SignalP-5.0
Deep TMHMM tool	25 April 2025	https://dtu.biolib.com/DeepTMHMM
NetPhos 3.1	25 April 2025	http://www.cbs.dtu.dk/services/NetPhos/
PROSITE database	25 April 2025	https://prosite.expasy.org/
MEME Suite	27 April 2025	http://memesuite.org/tools/meme
Phytozome databases	27 April 2025	https://phytozome.jgi.doe.gov/
iTOL server	27 April 2025	https://itol.embl.de/
PlantCARE database	28 April 2025	http://bioinformatics.psb.ugent.be/webtools/plantcare/html/
eggNOG-mapper	28 April 2025	http://eggnog5.embl.de/
WEGO 2.0	28 April 2025	https://wego.genomics.cn/
KEGG database	29 April 2025	https://www.kegg.jp/kegg/pathway.html
NPS@SOPMA	29 April 2025	https://npsa.lyon.inserm.fr/cgi-bin/npsa_automat.pl?page=/NPSA/npsa_sopma.html
UniProt	29 April 2025	https://www.uniprot.org/
STRING	30 April 2025	https://cn.string-db.org/
