# Supplementary material for: Genome-Wide Identification and Analysis of Chitinase GH18 Gene Family in Trichoderma longibrachiatum T6 Strain: Insights into Biocontrol of Heterodera avenae
Source: J Fungi (Basel). 2025 Oct 1;11(10):714. doi: 10.3390/jof11100714 (PMC12565608; doi:10.3390/jof11100714)
Supplement: Supplementary file 1 [file jof-11-00714-s001.zip › jof-3841329-GH18 Table S2.pdf]

Table S2 Primer sequences used in this study.

Target gene	Primer type	Sequence	Usage
Actin	Actin-F	CGTTCGTGACATCAAGGAGAA	qRT-PCR
	Actin-R	CGCTCTCAAGACCAAGGACA	
GH18-1	GH18-1F	CCGCCTCCAGTCTACGCACC	qRT-PCR
	GH18-1R	CCCCTTGTCCCCGTTCGCAT	
GH18-2	GH18-2F	ACCCCACCCCTACGCTTTGC	qRT-PCR
	GH18-2R	GCCATTCCAGCCCCAGGTGT	
GH18-3	GH18-3F	GTGTTAATGCCTTCACGCCC	qRT-PCR
	GH18-3R	CATCATGGCACCAGCAAAGG	
GH18-4	GH18-4F	GCTGCCCCTTCTGTCCTCCG	qRT-PCR
	GH18-4R	CCAGCCCATTTCCGAGGCGA	
GH18-5	GH18-5F	GAACTGCTCGTCGGAGGCGT	qRT-PCR
	GH18-5R	CTCGCCGCCATCCACCTCAA	
GH18-6	GH18-6F	CCAGTTCTTCGACCCGTCCC	qRT-PCR
	GH18-6R	ACGGTCGCGGGCTTTGTTG	
GH18-7	GH18-7F	CCTCCTCCGACAACCCTGCG	qRT-PCR
	GH18-7R	TGCCATTCTCCCACGAGCCC	
GH18-8	GH18-8F	CCGTGCCGGTGAGGAGGATG	qRT-PCR
	GH18-8R	GTGCTCCCGCCTCGACTCTG	
GH18-9	GH18-9F	CGAGGCCGTGCTCCAGATCC	qRT-PCR
	GH18-9R	ACCCGAGGATGGCAAAGGGC	
GH18-10	GH18-10F	CTACTTCGCGGCAACCCGGA	qRT-PCR
	GH18-10R	GGGCGCTGAGATGAAGCGGA	
GH18-11	GH18-11F	CGTCGTCTACTGGGGTGCCG	qRT-PCR
	GH18-11R	GATGGCGGGGATGAGCGAGG	
GH18-12	GH18-12F	TCCCCAACACAGAGCGCGAG	qRT-PCR
	GH18-12R	ACGACACCATCACGCCGGTT	
GH18-13	GH18-13F	TCTTGGCGATGAGTGGGCCG	qRT-PCR
	GH18-13R	CAAGCCCGGAAGCCTCGACA	
GH18-14	GH18-14F	TGCGGAGCCTGCTTGATGCT	qRT-PCR
	GH18-14R	GTGGGAGACACCGCAGCCAT	
GH18-15	GH18-15F	CCGCAGAACTGTGACGCCCT	qRT-PCR
	GH18-15R	GTCACCCCAGATCCACCGCC	
GH18-16	GH18-16F	AGCAACACATTTTCCTTCGC	qRT-PCR
	GH18-16R	TCCACTGGCTCTCTTCTCAA	
GH18-17	GH18-17F	TCCCCAACACAGAGCGCGAG	qRT-PCR
	GH18-17R	ACGACACCATCACGCCGGTT	
GH18-18	GH18-18F	GTGGTTCAGCACGCAAGCCG	qRT-PCR
	GH18-18R	CACTGCCCACCCTGCTCCTC	
